# Implementation of the 9th TNM for lung cancer: practical insights for radiologists

**DOI:** 10.1007/s00330-024-11345-8

**Published:** 2025-01-17

**Authors:** Gianluca Argentieri, Clara Valsecchi, Francesco Petrella, Lisa Jungblut, Thomas Frauenfelder, Filippo Del Grande, Stefania Rizzo

**Affiliations:** 1 Clinic of Radiology, Imaging Institute of Southern Switzerland (IIMSI), Ente Ospedaliero Cantonale (EOC), Via Tesserete 46, 6900 Lugano, Switzerland; 2 Department of Thoracic Surgery, Fondazione IRCCS San Gerardo dei Tintori, 20900 Monza, Italy; 3 Institute of Diagnostic and Interventional Radiology, University Hospital Zurich, Raemistrasse 100, 8091 Zurich, Switzerland; 4 Faculty of Biomedical Sciences, Università Della Svizzera Italiana (USI), Via G. Buffi 13, 6904 Lugano, Switzerland

**Keywords:** Lung neoplasm, Neoplasm staging, Prognosis, Radiology

## Abstract

**Abstract:**

Lung cancer is the most common and deadly cancer worldwide. The 9th edition of the tumor node meta (TNM) classification system, effective from January 1, 2025, introduces significant updates. Notably, the N2 category is newly divided into N2a (single-station involvement) and N2b (multiple-station involvement), which reflects distinct prognostic implications. Additionally, the M1c category is now subcategorized into M1c1 (multiple metastases in a single organ system) and M1c2 (metastases in multiple organ systems), affecting stage classification. This reclassification allows for potential downstaging, which could expand treatment options for affected patients. Accurate imaging remains crucial for the classification of anatomical stages. As the TNM system evolves, enhanced imaging precision will play a key role in implementing these updates and ultimately improve patient outcomes.

**Key Points:**

***Question**** The 9th TNM for lung cancer introduces changes in the N2 and M1c descriptors, to better align with new therapeutic options and outcome studies*.

***Findings**** Proper knowledge of the key changes of the 9th TNM can help radiologists offer clinicians a meaningful report*.

***Clinical relevance*** *Radiologists should incorporate the 9th TNM classification into their reports and discussions in multidisciplinary meetings, thus ensuring a common language across disciplines to enable clearer communication with other specialists, supporting more precise and cohesive decision-making in patient care.*

## Introduction

Lung cancer is the most incident cancer in the world, accounting for 2,480,675 new cases in both sexes and at all stages, as well as the most lethal, with 1,817,469 deaths in both sexes at all ages in 2022 [1].

The domain of lung cancer diagnosis and treatment has undergone substantial advancements in the last few years. Until a few years ago, effective interventions were primarily restricted to surgical resection and were reserved for early-stage tumors, whereas chemotherapy and radiotherapy were adopted for more advanced cancers. More recently, treatment options have progressed, leading to a growing number of applications of multimodal approaches throughout various stages of the disease. This includes the use of systemic therapies for early-stage cases, as well as localized treatments for more advanced stages [2]. Consequently, there has been a heightened emphasis on the anatomical extent of tumors in advanced stages, particularly regarding higher N and M categories.

The tumor node meta (TNM) is a fundamental tool for cancer staging, providing a universal nomenclature to describe the anatomical extent of the disease. Since its first introduction in 1987 by the American Joint Committee on Cancer (AJCC) and the Union for International Cancer Control (UICC) [3], the TNM classification system comprises three main elements: T, which indicates the extent of the primary tumor; N, which reflects lymph node involvement; and M, which denotes the presence of distant metastases. Each of these components is further divided into multiple categories (for instance, T1, T2) and subcategories (such as T1a, T1b). The combinations of T, N, and M classifications are then grouped into distinct stage categories, according to the International Association for the Study of Lung Cancer (IASLC) [4].

Effective January 1, 2025, the 9th edition of the TNM classification for lung cancer will be implemented [5–7], bringing several important updates from the 8th edition. Radiologists should be familiar with these changes to provide optimal information in radiological reports and contribute accurately during multidisciplinary meetings for clinical decision-making.

This review aims to highlight and critically evaluate the key changes in the 9th edition of the TNM system, helping to integrate these updates into radiology reporting.

## Key updates of the 9th edition of TNM

### Division of N2 category into N2a and N2b subcategories

**Fig. 1:**
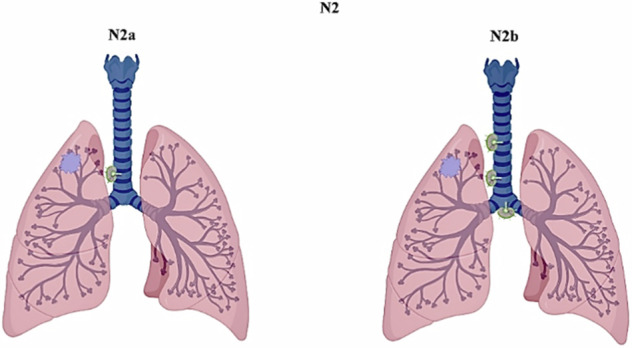
According to the 9th edition of TNM, the N2 (ipsilateral lymph nodes) is divided into single ipsilateral station (N2a) and multiple ipsilateral stations (N2b)

The N2 category in the 8th edition indicated the presence of metastases in ipsilateral mediastinal and/or subcarinal lymph nodes. In the 9th TNM, N2 is subdivided into single-station involvement (N2a) and multiple-station involvement (N2b) (Fig. 1). This update arises from recognizing a distinct and significant prognostic difference between these two groups, even when adjusting for variables like age, sex, histologic type, prior malignancy, geographic region, and resection completeness. Noteworthy, the 9th TNM quantifies nodal disease based on the number of affected stations rather than the total count of lymph nodes involved.

Despite the lack of detailed data on specific imaging techniques, biopsy methods, and their comprehensiveness, notable survival differences were observed in a large global database, underscoring the practical significance of these changes [5, 8, 9].

### Division of M1c subcategory into M1c1 and M1c2

**Fig. 2:**
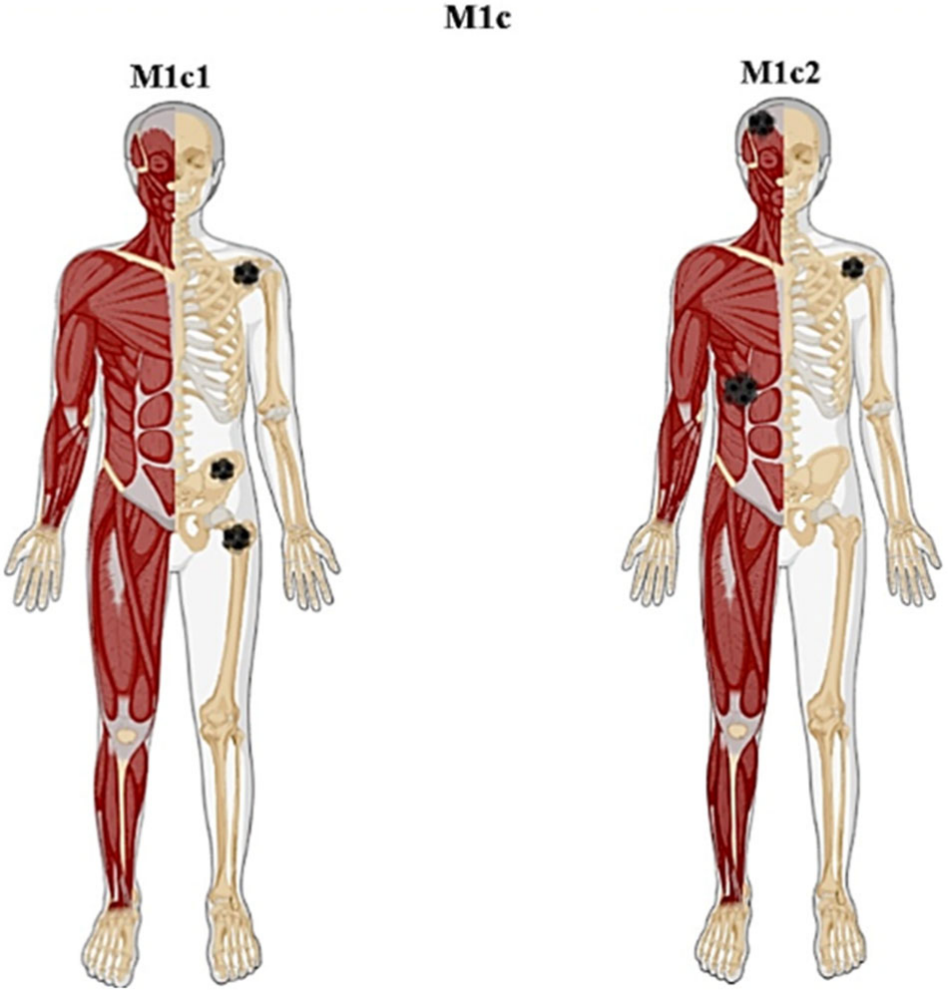
According to the 9th edition of TNM, the M1c (multiple extrathoracic metastases) is divided into metastases in a single organ/system (M1c1), including multiple bone lesions accounting for metastases in one organ, and metastases in multiple organ systems (M1c2)

The M1c category in the 8th edition indicated the presence of multiple extrathoracic metastases. In the 9th TNM, the M1c component is subdivided into multiple metastases in a single organ system (M1c1) and metastases in multiple organ systems (M1c2) [5–7] (Fig. 2).

While the distinctions among M1a and M1b categories are kept constant compared to the 8th edition, an analysis of combinations regarding the number of lesions and metastatic organ sites suggested that the most effective categorization should subcategorize the M1c category, according to the involvement of a single (M1c1) or multiple (M1c2) organ systems, respectively. Interestingly, assessments of metastatic burden according to size, number of lesions, and/or sites were inconsistent, with the sole exception of the number of organs/systems involved. Thus, the M1c1 definition should apply to an organ system, regardless of whether the organ is single, paired, or distributed throughout the body (such as the skeletal system). However, the 9th TNM does not define whether the M1c1 descriptor should impose a limit on the number of metastases within a single organ system.

## Revision of International Association for the Study of Lung Cancer (IASLC) stages according to the 9th TNM combinations

**Fig. 3:**
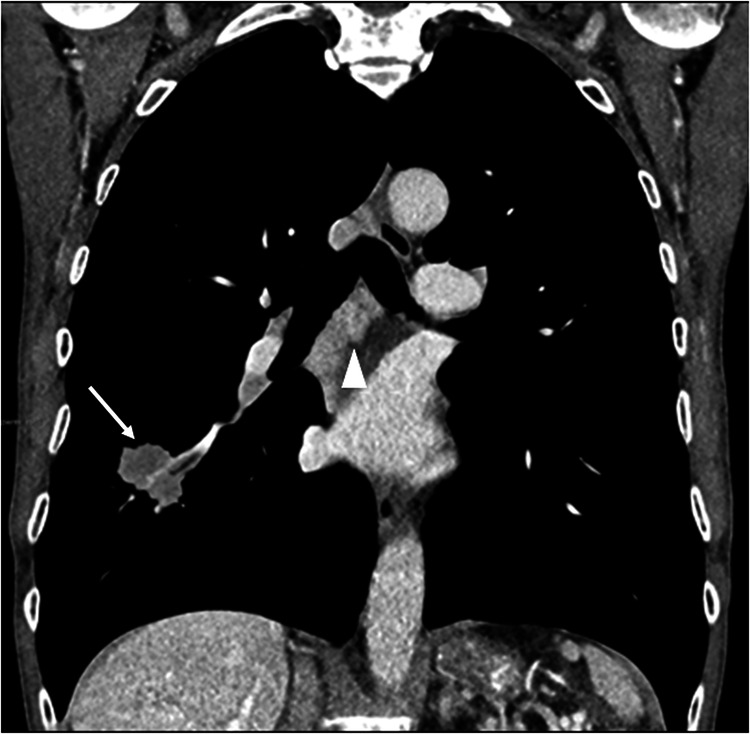
A 65-year-old male presented with a 3 cm lung adenocarcinoma (T1c) in the right lower lobe (white arrow) with single station subcarinal lymphadenopathies (arrowhead) (N2a). According to the 9th edition of TNM, the patient is classified as T1cN2aM0, corresponding to a stage IIB, whereas it would have been categorized as T1cN2M0 according to the 8th TNM, corresponding to a stage IIIA

**Fig. 4:**
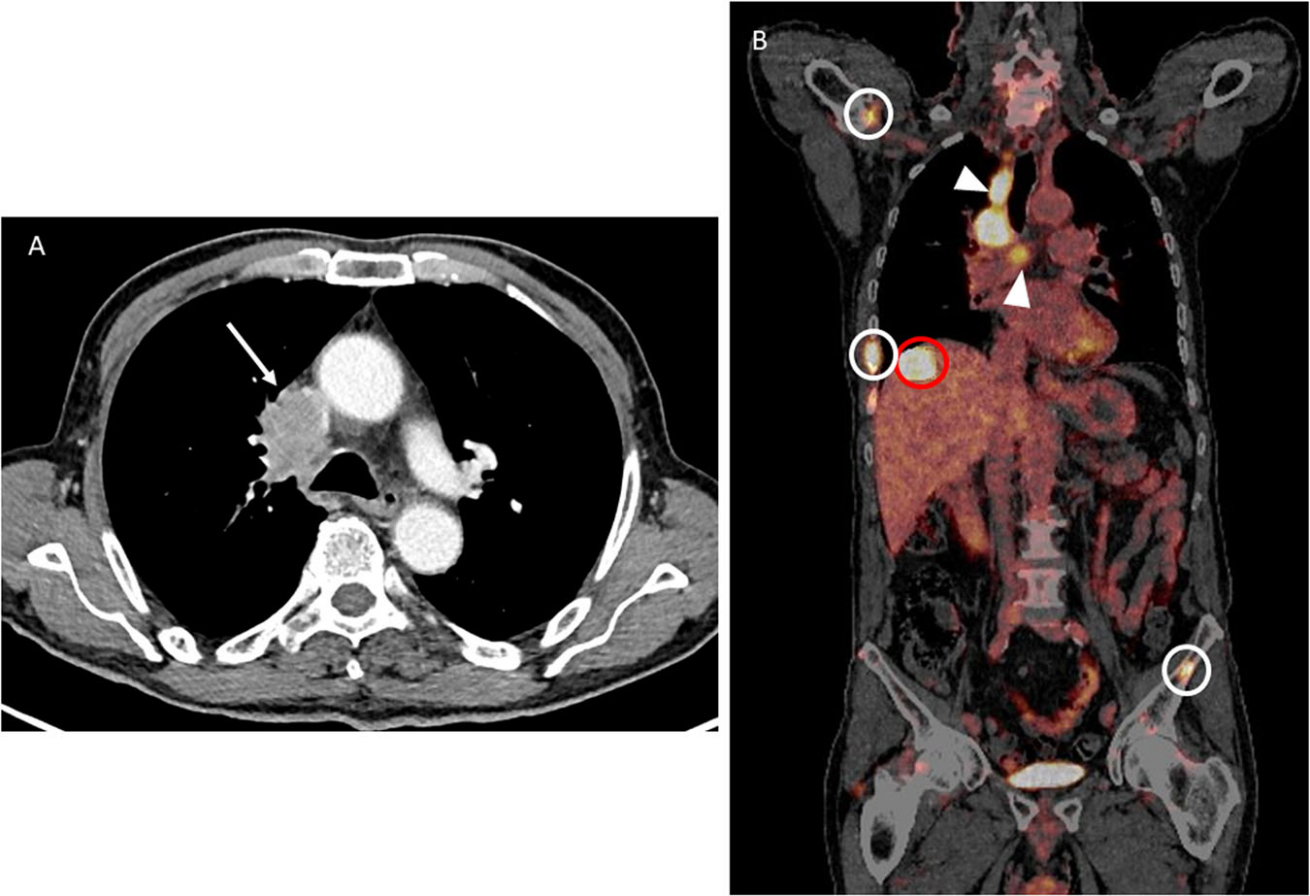
A 58-year-old male with a lung adenocarcinoma infiltrating the superior vena cava (T4) (white arrow in **A**). PET-CT (**B**) shows ipsilateral lymphadenopathies (white arrowheads) in superior paratracheal and subcarinal stations (N2b), as well as metastases in the liver (red circle) and bones (white circle) (M1c2), with a subsequent stage IVB (no stage changes according to the 9th TNM)

The abovementioned changes directly affect stage classification, with the subsequent reorganization of stages IIA, IIB, IIIA, and IIIB [8, 9] (Table 1). More precisely, the T1 N1 falls now in the IIA stage (IIB according to the 8th TNM), the T1 N2a falls in the IIB stage (Fig. 3), and the T1 N2b falls in the IIIA (both staged as IIIA according to the 8th TNM). Furthermore, the T2 N2a falls in the IIIA stage, and the T2 N2b falls in the IIIB (both staged as IIIA according to the 8th TNM); the T3 N2a falls in the IIIA stage, and the T3N2b falls in the IIIB (both staged as IIIB according to the 8th TNM); whereas the T4 N2a and the T4 N2b falls in the IIIB stage, as in the previous version of the TNM. The introduction of the 2 subcategories of M1c1 and M1c2 does not change the stage, which remains IVB (Fig. 4).

**Table 1:** Comparison of the International Association for the Study of Lung Cancer (IASLC) stages, according to the 8th and 9th editions of TNM, with highlighted changes in black boxes

The changes outlined above directly impact stage classification, resulting in a reorganization of stages IIA, IIB, IIIA, and IIIB [8, 9] (see Table 1). Specifically:

- T1 N1 is now classified as stage IIA (previously IIB in the 8th TNM).
- T1 N2a is now classified as stage IIB (Fig. 3).
- T1 N2b remains in stage IIIA (unchanged from the 8th TNM).
- T2 N2a is now classified as stage IIIA.
- T2 N2b is classified as stage IIIB (previously IIIA in the 8th TNM).
- T3 N2a remains in stage IIIA.
- T3 N2b is classified as stage IIIB (unchanged from the 8th TNM).
- T4 N2a and T4 N2b remain in stage IIIB, as in the prior version of TNM.

Additionally, the introduction of subcategories M1c1 and M1c2 does not alter the stage, which remains classified as IVB (Fig. 4).

These stage modifications primarily illustrate that higher-stage groups now consist of tumors with incrementally higher T or N categories, while the M category does not currently affect stage progression. Importantly, the addition of more subcategories within the N2 group has resulted in a certain downstaging of some lesions, potentially expanding treatment options. For example, a T1N2aM0 lesion previously classified as IIIA in the 8th TNM is now classified as IIB in the 9th edition. This downstaging highlights the increased therapeutic options available for lung cancer patients, even in cases previously deemed too advanced for surgical resection [2, 9].

## The 9th TNM in a radiological report

### Tumor (T)

The T category was not affected by the changes introduced by the 9th TNM edition.

Measurement of the maximum diameter is key for inclusion into the T1a (≤ 1 cm), T1b (> 1 cm and ≤ 2 cm), or T1c (> 2 cm and ≤ 3 cm) subcategories. The tumor size at imaging (c-stage) relies on the measurement of the solid component on thin-slice CT scans (≤ 1.5 mm slice thickness) using lung windows, without including the ground-glass component or lepidic component, if present. Multiplanar imaging may be utilized, when needed, to more accurately depict tumor size. If multiple solid nodules are present, the largest solid nodule should be used to determine the T category. Any direct extension of the primary tumor into an adjacent node is considered nodal involvement. As in the 8th TNM, T2 is subdivided into T2a (either tumor > 3 cm and ≤ 4 cm, and/or invasion of the visceral pleura/adjacent lobe, and/or involvement of the main bronchus or causing atelectasis/obstructive pneumonitis) and T2b (> 4 cm and ≤ 5 cm).

The T3 category includes tumors with a diameter > 5 cm and ≤ 7 cm; invasion of the parietal pleura or chest wall, thoracic nerves, or stellate ganglion; invasion of the pericardium, phrenic nerve, or azygos vein.

T4 includes tumors with any of the following features: > 7 cm invasion of the vertebra, lamina, spinal canal, subclavian vessels, brachial plexus, or cervical nerve roots; invasion of the thymus, trachea, carina, recurrent laryngeal nerve, esophagus, or diaphragm; invasion of heart or great vessels.

It is important to note that the classification of multiple nodules depends on their histological type and clinical characteristics. Multiple primary tumors, confirmed by distinct histological types, require a separate TNM classification for each. A separate tumor nodule—defined as an additional lesion with the same clinical and histological characteristics as the primary tumor—is categorized as T3 if located in the same lobe, T4 if in a different ipsilateral lobe, and M1a if in the contralateral lung.

### Node (N)

The 9th TNM continues to adopt and support the node definitions according to the IASLC Node Map, where lymph node stations are classified according to numbers (from 1 to 14) and sides (L for left, R for right) [4].

N0 (no regional lymph node metastases), N1 (metastases in ipsilateral pulmonary or hilar lymph nodes), and N3 (involvement of supraclavicular or scalene nodes or contralateral mediastinal/hilar nodes) categories have not been changed in the 9th TNM edition. The introduction of the N2a and N2b subcategories, indicating involvement of a single ipsilateral mediastinal/subcarinal nodal station, and involvement of multiple ipsilateral mediastinal or subcarinal nodal stations, respectively, underlies the importance of indicating in the radiology report the number of stations suspected, rather than the number of lymph nodes [10].

It is important to note that lymph nodes not specified in the N classification—such as diaphragmatic, intercostal, cervical, internal mammary, and axillary nodes—are classified under distant metastatic disease. CT is part of the standard work-up for staging lung cancer patients [11]. However, it is well recognized that CT’s ability to distinguish between positive and negative lymph nodes is limited, primarily relying on size criteria, even during follow-up [12, 13]. The introduction of node-RADS may enhance specificity in the future [14]. Due to CT’s low specificity, especially for enlarged nodes that may appear falsely positive in various inflammatory conditions such as sarcoidosis and sarcoid-like reactions, it is commonly supplemented by PET-CT [15]. In PET-CT, nodal involvement is assessed using a maximum standard uptake value (SUV) of 2.5 or by visually comparing the SUV to mediastinal background levels. A comparison of pooled sensitivity and specificity for nodal staging shows significantly higher values for PET-CT than CT alone (0.61 and 0.79 for CT; 0.85 and 0.90 for PET-CT, respectively) [16].

Given the limitations of imaging modalities alone in nodal staging, suspicious lymph nodes must undergo pathological confirmation when possible, particularly when curative treatment is intended. In fact, in the light of induction treatments based on chemotherapy and immunotherapy for potentially resectable lung cancers, a proper differential diagnosis between N2a and N2b disease might further contribute to better select patients maximally benefiting from surgical resection. In this scenario, imaging plays a crucial role in selecting biopsy targets to confirm the highest possible disease stage, often achieved through endobronchial ultrasound-guided fine needle aspiration (EBUS-TBNA). Target nodes are typically selected based on PET avidity [16–18].

### Metastases (M)

In the 9th TNM, the definition of M0 (no distant metastasis), M1a (malignant pleural or pericardial effusion or pleural/pericardial nodules, separate tumor nodules in a contralateral lobe), and M1b (single extrathoracic metastasis) categories are kept equal.

The distinction of multiple extrathoracic metastases (M1c) into involvement of a single organ/system (M1c1) or involvement of multiple organ/systems (M1c2) raises attention to consider a diffuse organ/system, such as the skeleton, as one organ. An organ system can be solitary (such as brain and liver), paired (such as adrenals or kidneys), or diffuse throughout the body (such as bone), in the 9th TNM for the lung.

The progressive addition of subcategories in the M1 classification—ranging from two in the 7th TNM to three in the 8th and four in the 9th—underscores the significance and complexity of accurately defining oligometastatic disease. Ultimately, the oligometastatic burden lies on a continuum without a clear inflection point, making it most appropriately assessed through clinical trials and expert judgment. A checklist of all the relevant information to be included in the report for staging according to the 9th TNM is summarized in Table 2.

**Table 2:** Reporting checklist to ensure that all the relevant information is included in the report

TNM category	Features to report
T (tumor)	-Maximum diameter of the solid component (thin-slice CT, ≤ 1.5 mm, lung windows) -Tumor extension (indicate all involved areas): -Visceral pleura -Main bronchus -Carina -Adjacent lobe -Chest wall -Pericardium -Phrenic nerve -Azygos vein -Separate ipsilateral nodules (specify location: ______)
N (nodes)	-Number of involved nodal stations -Location of nodal stations (IASLC Node Map [4]): -Ipsilateral mediastinal (specify single station/multiple stations to comply with 9th TNM) -Contralateral mediastinal -Subcarinal -Supraclavicular
M (metastases)	-Presence and location of distant metastases -Malignant pleural or pericardial effusion and/or nodules -Tumor nodules in the contralateral lung -Single/multiple extrathoracic metastases, in single or multiple organ/systems (consider multiple metastases in a single/paired organ as single system metastasis to comply with the 9th TNM)

## Conclusion

The 9th edition of the TNM classification for lung cancer introduces a more refined and detailed approach to anatomical staging, designed to align with new therapeutic options and insights from various outcome studies. While anatomical characteristics remain essential for local treatments, non-anatomical tumor factors—such as driver mutations and PD-L1 expression—are increasingly pivotal in guiding systemic therapies. Future staging systems may incorporate these non-anatomical elements, prompting ongoing adjustments to anatomical staging in a progressive cycle.

It is essential that radiologists incorporate the new 9th edition TNM classification into their reports and discussions in multidisciplinary meetings, ensuring a common language across disciplines. This alignment enables clearer communication with other specialists, supporting more precise and cohesive decision-making in patient care.

## Abbreviations

IASLC: International Association for the Study of Lung Cancer
SUV: Standard uptake value
TNM: Tumor node metastases
